# Reconfigurable radiofrequency filters based on versatile soliton microcombs

**DOI:** 10.1038/s41467-020-18215-z

**Published:** 2020-09-01

**Authors:** Jianqi Hu, Jijun He, Junqiu Liu, Arslan S. Raja, Maxim Karpov, Anton Lukashchuk, Tobias J. Kippenberg, Camille-Sophie Brès

**Affiliations:** 1 École Polytechnique Fédérale de Lausanne, Photonic Systems Laboratory (PHOSL), STI-IEL, Station 11, 1015 Lausanne, Switzerland; 2 École Polytechnique Fédérale de Lausanne, Laboratory of Photonics and Quantum Measurements (LPQM), SB-IPHYS, Station 3, 1015 Lausanne, Switzerland

**Keywords:** Microwave photonics, Frequency combs

## Abstract

The rapidly maturing integrated Kerr microcombs show significant potential for microwave photonics. Yet, state-of-the-art microcomb-based radiofrequency filters have required programmable pulse shapers, which inevitably increase the system cost, footprint, and complexity. Here, by leveraging the smooth spectral envelope of single solitons, we demonstrate microcomb-based radiofrequency filters free from any additional pulse shaping. More importantly, we achieve all-optical reconfiguration of the radiofrequency filters by exploiting the intrinsically rich soliton configurations. Specifically, we harness the perfect soliton crystals to multiply the comb spacing thereby dividing the filter passband frequencies. Also, the versatile spectral interference patterns of two solitons enable wide reconfigurability of filter passband frequencies, according to their relative azimuthal angles within the round-trip. The proposed schemes demand neither an interferometric setup nor another pulse shaper for filter reconfiguration, providing a simplified synthesis of widely reconfigurable microcomb-based radiofrequency filters.

## Abstract (web summary)

For microcomb-based radiofrequency filters pulse shapers are required, which increase the system cost, footprint, and complexity. Here, the authors bypass this need by exploiting versatile soliton states inherent in microresonator and achieve reconfigurable radiofrequency filters.

## Introduction

Owing to the ever-maturing photonic integration, radiofrequency (RF) photonic systems and subsystems have been brought to new height^1–5^, in terms of footprint, scalability, and potentially cost-effectiveness. Particularly, RF filtering towards chip-scale is a key enabling function^6–17^. Paradigm demonstrations include the integration of the basic filtering blocks, such as delay lines^7^, optical spectral shaper^8^, programmable mesh topologies^9^, and ring resonators^10–12^, as well as the use of stimulated Brillouin scattering (SBS) in waveguides^13^. Recently, an all-integrated RF photonic filter has been shown in a monolithic platform^14^. Among others, RF filters constructed from tapped delay line (TDL) structures attract great attention. They can be classified into two types^18^, depending on whether the filtering profile is given by the physical path delays^9,14^ or the light source spectra^7,8,15–17,19–22^. While the former approach is straightforward, a multiwavelength source combined with dispersive propagation also functions as a TDL filter. This greatly simplifies the structural complexity of a finite impulse response filter, as only a single dispersive delay line is needed. Nevertheless, the main complexity is then shifted to the multiwavelength source. Electro-optic (EO) combs^8,19,20^ or mode-locked lasers^21,22^ are generally adopted as light sources, which remain expensive and bulky options.

Integrated optical Kerr combs (microcombs) have appeared as an interesting alternative. Microcombs have already been applied not only to filter RF signals^15–17^ but also for various RF photonic processing, such as true-time delay beamforming^23^, RF channelization^24^, and analog computation^25^. The large comb spacing of microcombs also enhances RF filters with broader Nyquist zone (spur-free range), lower latency^15^, and less dispersion-induced fading, as well as larger number counts of equivalent delay lines^16^, unparalleled by other approaches. However, so far all these microcomb-based RF filters have been implemented on either dark pulses^15,26^ or complex soliton crystal states^16,17^. Additional programmable pulse shaping modules are inevitably required to equalize or smooth the comb spectral shape. Thus, the system complexity is significantly increased while the potential for low-cost and high-volume applications is compromised. To date, harnessing the smooth sech^2^ spectral envelope of single soliton^27–29^ or other regulated dissipative Kerr soliton (DKS) states for photonic RF filtering is yet to be investigated. Because single-soliton microcombs have facilitated a myriad of applications, ranging from Tb/s coherent communication^30^, ultrafast and long-distance ranging^31,32^, dual-comb spectroscopy^33^, and astronomical spectrometer calibration^34^, to microwave synthesis^35^, a great benefit for RF filtering can be expected.

In this paper, we demonstrate soliton microcomb-based RF photonic filters without any external pulse shaping. In addition, the synthesized RF filters can be all optically reconfigured through the internal versatile soliton states. Specifically, we trigger, in a deterministic fashion, the perfect soliton crystals (PSCs) to multiply the comb spacing^36,37^, thereby dividing the RF passband frequencies. Moreover, we achieve filter reconfiguration based on versatile two-soliton microcombs (TSMs). The spectral interference of two solitons is functionally equivalent to an interferometric setup, shifting the filter passband frequency via modification of the angle between them. A proof-of-concept filter reconfiguration experiment is also shown using TSM-based RF filters. The internal exploitation of abundant and regulated soliton formats of microresonator effectively bypasses the need of another programmable pulse shaper and interferometric setup for RF filter tuning^15,16^. Thus, the proposed scheme dramatically reduces the system complexity and form factor of microcomb-based RF filters, and are readily applicable to the current radar systems, 5G wireless, and satellite communications.

## Results

### Principle of soliton microcomb-based RF filters

**Fig. 1:**
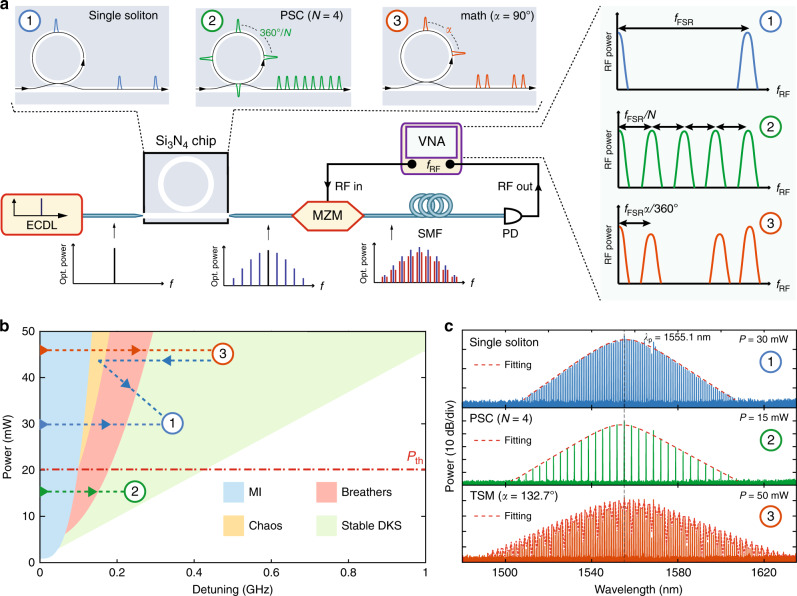
Schematic diagram of reconfigurable soliton-based radiofrequency photonic filters and their underlying microcomb generation. **a** The conceptual setup consists of four parts: microcomb generation, radiofrequency (RF) signal upconversion, dispersive propagation, and photodetection. ECDL external cavity diode laser, MZM Mach–Zehnder modulator, SMF single-mode fiber, PD photodiode, VNA vector network analyzer. Various RF filters are synthesized based on versatile soliton microcombs: (1) single-soliton-based RF filter with a passband centered at *f*_FSR_ (blue); (2) *N* − PSC- (perfect soliton crystals of *N* equally spaced solitons within one round-trip) based RF filters with a passband centered at *f*_FSR_/*N* (green, *N* = 4 is shown); (3) two-soliton microcomb (TSM)-based RF filters with a passband centered at *f*_FSR_*α*/360° (orange), where *α* is the relative azimuthal angle between two solitons (*α* = 90° is shown). **b** Simulated stability diagram of the Lugiato–Lefever equation (LLE) involving the experimental avoided mode crossing (AMX) condition. Four different stability regions are listed: modulation instability (MI, blue), breathers (red), spatio-temporal and transient chaos (chaos, yellow), and stable dissipative Kerr soliton (DKS, green). PSC and TSM/single-soliton spectra are obtained by distinct approaches. PSC states are accessed under the threshold power to avoid the chaos region. Single-soliton or TSM states are accessed above the threshold power, by either directly falling to the states or backward tuning from a higher number of solitons. **c** Examples of experimentally generated spectra at resonance of 1555.1 nm: (1) single-soliton, (2) PSC (*N* = 4), and (3) TSM (*α* = 132.7°) with envelope fitting. The pump power is also shown for each microcomb generation.

 Figure 1a illustrates the conceptual setup for soliton microcomb-based RF filters. First, a telecom C-band continuous wave (CW) laser initiates microcomb generation, where each comb line serves as the RF filter tap. By modulating the RF signals from a vector network analyzer (VNA) on an electro-optic Mach–Zehnder modulator (MZM), the RF signals are broadcast to each microcomb mode. Then, the upconverted signals are propagated through a spool of single-mode fiber (SMF) to acquire incremental delay between filter taps. Finally, the signals are converted back to the RF domain in a fast photodetector (PD). The detailed experimental setup is described in the “Methods”. This arrangement exactly corresponds to a TDL filter, where the power of each comb line *p*_*k*_ is the tap weight, and the delay is determined by the comb spacing *f*_*m*_ and the accumulated dispersion *ϕ*_2_ = −*β*_2_*L* (the product of SMF second-order dispersion *β*_2_ and fiber length *L*). When the filter tap weights take the sech^2^ envelope of a single-soliton comb (case 1), the RF filter response is given as (see Supplementary Note 1):

1 $$H({f}_{{\rm{RF}}}) \sim \cos (2{\pi }^{2}{\phi }_{2}{f}_{{\rm{RF}}}^{2})\mathop{\sum }\limits_{n=-\infty }^{\infty }G({f}_{{\rm{RF}}}-n{f}_{{\rm{FSR}}}),$$

where *f*_FSR_ and *G*(*f*_RF_) are, respectively, defined as 1/(2*πϕ*_2_*f*_*m*_) and $2\frac{T}{{T}_{0}}\frac{{f}_{{\rm{RF}}}}{{f}_{{\rm{FSR}}}}$sinh${}^{-1}(\frac{T}{{T}_{0}}\frac{{f}_{{\rm{RF}}}}{{f}_{{\rm{FSR}}}})$, with *T* = 1/*f*_*m*_ the repetition period, and *T*_0_ the soliton pulse width. *f*_RF_ denotes the RF frequency. Note that higher-order dispersion of SMF is neglected here to give a more intuitive picture. The overall RF filter response can be seen as a periodic function of lineshape *G*(*f*_RF_) with RF free spectral range (FSR) of *f*_FSR_, modulated by an envelope due to the double-sideband (DSB) modulation scheme being used. As the tap weights are all positive, the passband frequencies of the RF filters are at every multiple of *f*_FSR_, including a DC response. Throughout this article, we focus on the first passband.

Besides, by exploiting the rich soliton states of microresonator, the RF filters can be easily reconfigured at no additional cost nor complexity. Among soliton crystal structures^38,39^, the defect-free PSC is of particular interest, as *N*(*N* ∈ *N*_+_∣*N* ≥ 2) equally spaced solitons (case 2) within one round-trip time simply multiplies the initial comb spacing by *N* times. This imparts *N* times division of the filter passband frequencies while preserving the filter bandwidth. The automatic PSC control is equivalent to the Talbot-based processer for discrete programming the RF filters in ref. ^22^. Less intuitively, all-optical reshaping of the RF filters can also be achieved via versatile TSM spectra (case 3). Two solitons residing in one period induce sinusoid interference on the sech^2^ spectral shape of a soliton, modulating the tap weights of the TDL filter. This rewrites the RF filter response as (see Supplementary Note 1):

2 $$H({f}_{{\rm{RF}}}) \sim 	\, \cos (2{\pi }^{2}{\phi }_{2}{f}_{{\rm{RF}}}^{2})\left[\mathop{\sum }\limits_{n=-\infty }^{\infty }2G({f}_{{\rm{RF}}}-n{f}_{{\rm{FSR}}})\right.\\ 	\, +G\Big({f}_{{\rm{RF}}}-\Big(n-\frac{\alpha }{2\pi }\Big) {f}_{{\rm{FSR}}}\Big)+G \Big({f}_{{\rm{RF}}}-\Big(n+\frac{\alpha }{2\pi }\Big){f}_{{\rm{FSR}}}\Big)\Bigg],$$

where *α* is the relative azimuthal angle between two solitons (expressed in radian for calculation). Clearly, new RF passbands of halved amplitude appear due to two-soliton interference, which is displaced at both sides from the initial response according to the azimuthal angle between them. Thus, the RF filter passbands can slide inside *f*_FSR_ by modifying the relative soliton angles. Unlike ref. ^16^ in which the authors artificially introduce the sinusoidal modulation via programming the spectral carving, we alleviate the need for a pulse shaper and realize sinusoidal modulation by directly generating a series of TSM spectra. This novel scheme achieves wideband reconfiguration of RF filters without either interferometric configuration or additional pulse shaping.

### Experimental implementation

The soliton microcombs used for RF filtering are generated from a 103.9 GHz ultra-low loss integrated silicon nitride (Si_3_N_4_) microresonator (*Q* ~ 1 × 10^7^), fabricated by the photonic Damascene reflow process^40^. By employing the frequency-comb-assisted diode laser spectroscopy, the detailed properties of resonances and the integrated group velocity dispersion of the microresonator are measured (see Supplementary Note 2). Strong avoided mode crossings (AMXs) are observed ~1565 nm, which lead to the modulation of intracavity CW background, thereby resulting in the ordering of the DKS pulses^41^ and the formation of soliton crystals^36,38,39^. Figure 1b shows the simulated stability diagram (see “Methods”), which consists of modulation instability (MI), breathers, chaos (spatio-temporal chaos and transient chaos), and stable DKS states. In addition, it has been revealed that the pump power level is critical for whether the PSCs or stochastic DKS states are formed^36^ (see Supplementary Note 3). In our case, the threshold pump power *P*_th_ is found to be ~20 mW in the bus waveguide. When the laser scanning route is operated below threshold pump power, PSC states can be accessed without crossing the chaos region. Contrarily, DKS states with stochastic soliton number are accessed above the threshold power. Experimentally, the single-soliton and TSM states are obtained by either directing falling to the states or backward tuning from the states with higher soliton number^42^. Note that switching from PSC to single soliton or TSM is prohibited unless the pump power is increased to be over the threshold^36^. Thus, through controlling the pump power and resonance frequency, various soliton microcombs (single soliton, PSC, and TSM) can be obtained on demand to produce the desired RF filter responses. For example, Fig. 1c shows three distinct optical spectra obtained from the resonance of 1555.1 nm: single soliton, PSC (*N* = 4), and TSM (*α* = 132.7°, see “Methods”), respectively. It is also worthwhile to mention that although this type of RF filter does not require coherent comb states, the high-intensity noise of MI combs is certainly undesirable^15^. As the intensity noise will be transferred to the synthesized RF filters, mode-locked comb states are preferred to minimize the noise at the RF output.

**Fig. 2:**
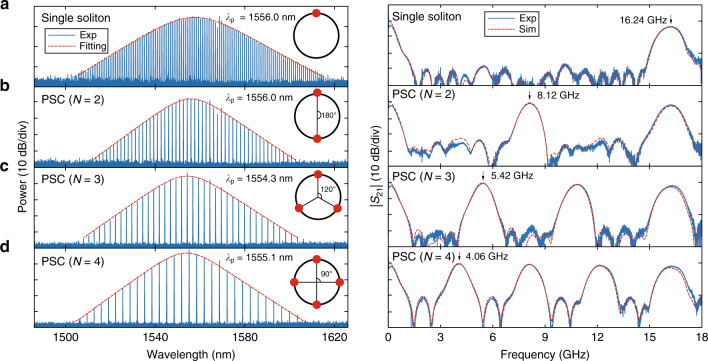
Single-soliton/PSC spectra and their corresponding RF photonic filters. Through deterministic accessing PSC states of different resonances, the RF filter passbands can be divided correspondingly. Left column: microcomb spectra (blue: experiment; red: sech^2^ fitting); right column: corresponding normalized RF filter responses (blue: experiment; red: simulation). **a** RF filter centered at 16.24 GHz based on single soliton from resonance of 1556.0 nm; **b**–**d** RF filters centered at 8.12, 5.42, and 4.06 GHz based on two , three , four times PSC, generated at resonances of 1556.0, 1554.3, and 1555.1 nm, respectively. The insets of left column illustrate soliton distribution inside the microresonator: **a** single-soliton; **b**–**d** equally spaced solitons (PSCs) with adjacent angles of 180°, 120°, and 90°, respectively (360°/*N*, *N* = 2, 3, 4).

 Figure 2 depicts the RF photonic filters using single soliton and various PSC microcombs. The single-soliton-based RF filter (Fig. 2a) is centered at 16.24 GHz, with main-to-sidelobe suppression ratio (MSSR) of 23.2 dB. Further, various PSC states are deterministically obtained at different resonances under the threshold power, thereby all optically reconfiguring the corresponding RF filters. The comb spacing multiplication via PSC results in the division of the corresponding RF passbands. RF filters centered at 8.12, 5.42, and 4.06 GHz (Fig. 2b–d) are experimentally synthesized through 2, 3, and 4 equally spaced solitons, with MSSR of 22.6, 25.6, and 20.4 dB, respectively. All these RF filters achieve MSSR over 20 dB without additional programmable spectral shaping. The MSSR here is limited by the smoothness of the optical spectra^19^, as several AMX can be seen in the microcombs. Nevertheless, all these microcombs preserve well the sech^2^ envelope, and remained smooth after amplification. In addition, the measured RF filter responses are in excellent agreement with simulations, by taking into account of third-order dispersion (*β*_3_) of SMF (see “Methods”). Note that the bandwidths of experimentally synthesized RF filters broaden slightly with their center frequencies, also due to the third-order dispersion of SMF^15^.

**Fig. 3:**
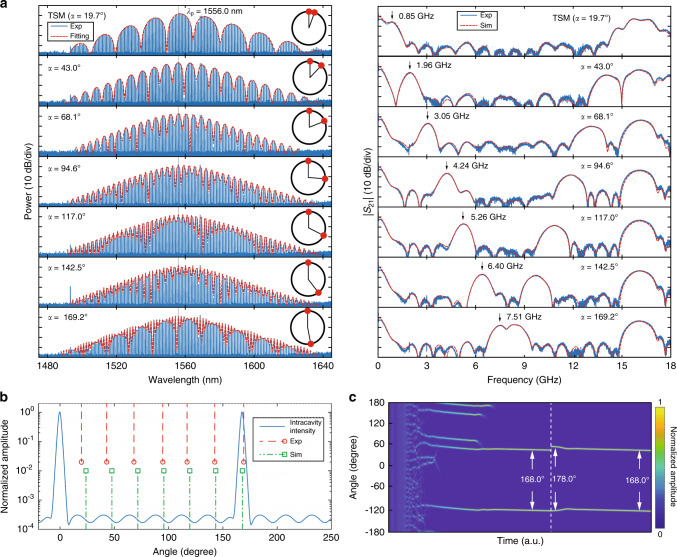
TSM spectra and their corresponding RF photonic filters, together with TSM simulation investigation. By accessing different two-soliton states, the RF filters can be all optically reconfigured. **a** Left column: TSM spectra at resonance of 1556.0 nm (blue: experiment; red: envelope fitting). The insets illustrate two-soliton distribution inside the microresonator: the angles between them are 19.7°, 43.0°, 68.1°, 94.6°, 117.0°, 142.5°, and 169.2°, respectively. Right column: corresponding normalized RF filter responses (blue: experiment; red: simulation) with passbands at 0.85, 1.96, 3.05, 4.24, 5.26, 6.40, and 7.51 GHz, respectively. **b** Simulation of TSM relative azimuthal angles. One example of the simulated TSM intracavity intensity profile (blue), where AMX-induced background modulation is observed. The red and green lines, respectively, indicate the measured and simulated possible azimuthal angles between two solitons. **c** Simulation of the intracavity waveform evolution of TSM for robustness test. First, TSM state with a relative angle of 168.0° is excited by scanning the pump over the resonance. Once the TSM becomes stable, a 10.0° perturbation is introduced to one of the solitons at white dashed line. The relative angle will re-stabilize to the original angle of 168.0° after a period of free running.

 Figure 3a shows the TSM spectra and their corresponding RF filter responses, pumped at resonance of 1556.0 nm. According to Eq. (2), the first passband frequencies of RF filters scale linearly with the relative angles between two solitons, so that the filter reconfiguration is achieved. In the experiment, TSM spectra with relative angles of 19.7°, 43.0°, 68.1°, 94.6°, 117.0°, 142.5°, and 169.2° are obtained, where the angles are extracted from the fitting of the microcomb spectral envelope (see “Methods”). The measured RF filters are correspondingly centered at 0.85, 1.96, 3.05, 4.24, 5.26, 6.40, and 7.51 GHz, confirming the linear relation with the soliton angle (see Supplementary Note 4). As in the case of PSC, a slight broadening of the filter passband width from 490 to 620 MHz is attributed to the third-order dispersion of SMF. Overall, the RF filters obtained at resonance 1556.0 nm could vary from DC to 8.1 GHz (*f*_FSR_/2) with a maximum grid of 1.2 GHz, while roughly preserving the filter bandwidth in the meantime. The granularity of TSM-based RF filters can be further reduced to <1 GHz by exploiting adjacent resonances of 1556.0 nm (see Supplementary Note 4). Also, mirrored passband responses of the TSM-based RF filters coexist between 8.1 and 16.2 GHz.

Importantly, the possible angles between two solitons are determined by the overall AMX profile, and are rather robust to both laser power and frequency detuning, thereby deterministically dictating the filter passband frequencies to be either one of those shown in Fig. 3a. To gain insights into the relative angles between two solitons, we also perform perturbed Lugiato–Lefever equation (LLE) simulation to investigate the TSM formations (see “Methods”). The blue curve in Fig. 3b shows one example of the steady-state two-soliton temporal intracavity profile. Due to the AMX effect, periodic intensity modulation is observed upon the CW background. It is clearly seen that the soliton can only be excited at specific parameter gradients^41^, as manifested by the green dashed lines, which correspond to the stationary solutions obtained in the simulation. These possible soliton angles are in good agreement with the experimental results, which is indicated as red dashed lines. To further test the robustness of the angle between two solitons, an external perturbation is deliberately introduced on their relative angle. Figure 3c illustrates the dynamical evolution of the two-soliton formation. The simulation is initiated as a standard laser scanning scheme to kick out two solitons. Once the simulation reaches a stable two-soliton solution (relative angle of 168.0°), one of the solitons is dragged from its original position by a 10.0° on purpose. After a period of free-running, the two solitons converge back to their original relative positions, again at 168.0° apart. This confirms the regulation of two solitons under AMX background modulation.

### RF filter reconfiguration experiment

**Fig. 4:**
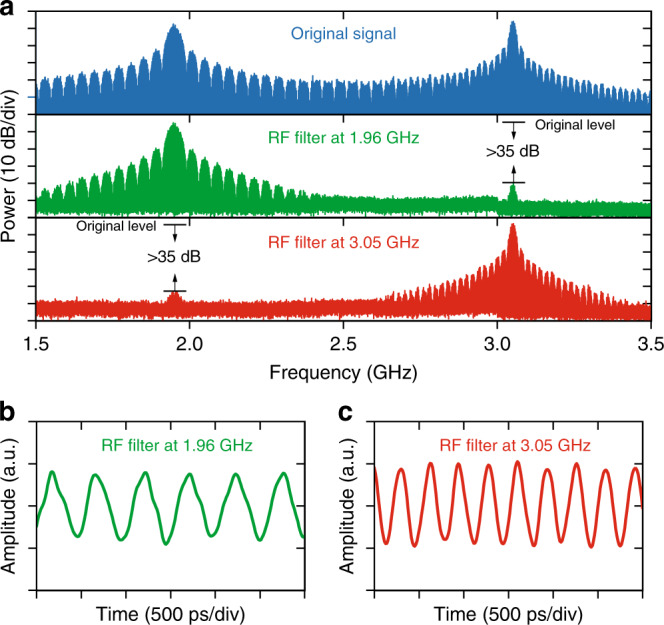
Proof-of-concept experiment using TSM-based RF filters. Two PSK signals with 40 Mb/s modulation at 1.96 GHz and 20 Mb/s modulation at 3.05 GHz are filtered by the TSM-based RF filters centered at 1.96 and 3.05 GHz, respectively. **a** From top to bottom: electrical spectra of original microwave signal, signal after 1.96 GHz filter, and signal after 3.05 GHz filter. **b** Waveform after 1.96 GHz filter. **c** Waveform after 3.05 GHz filter.

A proof-of-concept RF filter reconfiguration experiment is also illustrated in Fig. 4 using TSM-based RF filters (see “Methods”). Two superimposed phase-shift keying (PSK) signals in which a 40 Mb/s modulation at 1.96 GHz tone and a 20 Mb/s modulation at 3.05 GHz tone are prepared as input test signals. The RF filters are then, respectively, reconfigured at 1.96 and 3.05 GHz to filter the input signals, by triggering the TSM spectra of corresponding soliton angles. At the output of the RF filters, the nearly complete rejection of either one of the PSK signals is observed on the electrical spectra (Fig. 4a), where the extinction ratios exceed 35 dB for both cases. Figure 4b, c shows the filtered output RF waveforms. The periodicity of the output temporal traces corroborates the filtering of the original RF signals.

## Discussion

A comparison of the soliton RF filters presented in this work with other RF photonic filters based on various multiwavelength sources is shown in Table 1. First, we see that the MSSR achieved by soliton microcomb is comparable or even better than other implementations that do not require pulse shapers, such as combs based on cascaded modulators^19^, laser arrays^7^, or mode-locked lasers^21,22^, while none of them is integrated solutions regarding the light sources. Second, all of the past microcomb-based RF filters require, to our knowledge, two high-fidelity pulse shapers^15–17^, in order to equalize the largely unbalanced comb line intensity in the complex microcomb states. The achievable MSSR is indeed higher as expected, due to the fine equalization of comb amplitudes. Without pulse shapers, these approaches are not able to achieve similar MSSR nor tunability. In our case, the MSSR is mainly limited by the spectral roughness induced by mode crossings. One could envision combining our current implementation with an integrated spectral shaper as previously demonstrated^8^, although the device is based on indium phosphide platform. The integrated shaper can enhance our filters with improved MSSR and faster reconfiguration, while the soliton microcombs can greatly simplify the EO combs incorporating seven cascaded modulators used in ref. ^8^. It is worth noting that using integrated shaper for comb equalization in complex microcomb spectra would be inefficient, due to the shaper’s limited extinction ratio. Thus, the ability to control the soliton states among microcombs is truly essential for practical implementation.

**Table 1:** Comparison of RF photonic filters based on multiwavelength sources.

References	Light source	Pulse shaper required	MSSR	Passband frequency	Gain	Noise figure	Photonic integration
Ref. ^22^	Mode-locked laser	N	20.4 dB	0.6–1.8 GHz	N/A	N/A	*N*
Ref. ^21^	Mode-locked laser	N	24 dB	8–16 GHz	N/A	N/A	*N*
Ref. ^7^	Laser array	N	10 dB	0–50 GHz	N/A	N/A	Dispersive delay line
Ref. ^19^	EO comb, four modulators	N	28.2 dB/61 dB w/wo nonlinear shaping	0.8–10.4 GHz	−40 dB	N/A	*N*
Ref. ^20^	FP-EO comb, one modulator	Y, one	30 dB	2.6–13.9 GHz	N/A	N/A	*N*
Ref. ^43^	EO comb, four modulators	Y, one	32 dB	2–8 GHz	0 dB	24 dB	*N*
Ref. ^8^	EO comb, seven modulators	Y, two	35 dB	0.6–4 GHz	−3 dB	N/A	Spectral shaper
Ref. ^15^	Microcomb	Y, two	25 dB	2.5–17.5 GHz	N/A	N/A	Microresonator
Ref. ^16^	Microcomb	Y, two	48.9 dB	1.4–11.5 GHz	N/A	N/A	Microresonator
Ref. ^17^	Microcomb	Y, two	N/A	3.2–19.4 GHz	N/A	N/A	Microresonator
This work	Microcomb	N	25.6 dB	0.8–16.2 GHz	1.6 dB	29.8 dB	Microresonator

The best values of parameters extracted from the references are used in the table.

*Y* yes, *N* no, *N/A* not available, *EO* electro-optic, *FP* Fabry–Perot.

From the table, it is also evident that only a few past works report the link performances of comb-based RF filters. We carried out simple optimization of the RF filter link specifically based on 4-PSC as an example, where we achieve simultaneous positive link gain and noise figure within 30 dB (see Supplementary Note 5). Although the link performance is not yet as good as that of pure microwave solutions, it is already very close to the optimized performances of the state-of-the-art comb-based RF filters^43^. In addition, we achieve widely reconfigurable RF photonic filters from 0.8 to 16.2 GHz, taking into account both passbands obtained from TSM spectra. This multioctave filter operation is generally considered challenging by pure RF engineering^44,45^, especially at higher carrier frequency towards millimeter wave or terahertz wave. However, there is no such limitation for microwave photonic filters. It is worth mentioning the recent work on RF photonic filters synthesized from passive cascaded ring resonators with a wide continuous tuning capability^12^. However, in order to configure the filters, a number of power-consuming thermal heaters have to be controlled with fine resolution voltage sources. Although our filters cannot be tuned continuously, the exploration of multiple resonances dramatically enriches the achievable RF filters, while the possible controlled mode interaction^46^ or using dichromatic pumps^47^ may empower our filters with continuous tuning ability (see Supplementary Note 6). Moreover, additional functionalities, such as photonic downconversion of microwave signals, can be simultaneously realized in the microcomb-based filter setup, without electrical mixer and local oscillators^48^. The microcomb FSR will act as the local oscillator, which is promising for on-chip terahertz wave signal processing.

The RF filters obtained in this study are based on microcombs generated in a 103.9 GHz microresonator. If one targets RF filters with narrower bandwidth, a microresonator with larger size generating smaller FSR comb will be a better choice, as the filter bandwidth scales with the comb FSR given a certain soliton pulse width (see Supplementary Note 1). The recent ultra-low loss Si_3_N_4_ technology has allowed soliton formation down to the 10 GHz range^35^, which would enable RF filters of only tens of MHz bandwidth without additional pulse shaping. However, in order to maintain the filter center frequency when using small FSR combs, longer dispersive fiber is needed, while the envelope fading becomes more severe under the DSB modulation scheme^20^. In this case, more sophisticated modulation schemes are required, such as single-sideband modulation with broadband electrical 90° hybrid, in order to eliminate the fading effect. In comparison, microcombs of large comb spacing allow for a simple RF uploading method. Also, since we are interested in RF filters up to ~16 GHz (limited by the measurement range of VNA), the underlying combs with repetition rate over 32 GHz are preferred to avoid spurs generated from the signal beating with other comb lines^19^. Since obtaining a narrow RF photonic filter is not the primary interest in the current study, we utilize the 103.9 GHz microresonator in the experiment. The techniques presented here are compatible with other microcomb FSR.

In summary, we demonstrate reconfigurable soliton-based RF photonic filters using simplified approaches. Contrary to previous demonstrations where pulse shapers are necessary to obtain descent passband responses^15–17^, the proposed schemes are intrinsically well shaped with the smooth spectral envelopes of solitons. More importantly, we harness various intrinsic DKS states of microresonator, like PSC and TSM, for RF filter reconfiguration at no additional cost. The diversity and regularization of soliton formats in microresonator are investigated in the favor of RF photonic filters. To a certain extent, these inherent soliton states could be in place of substantial efforts made in the past for reconfiguring the comb-based RF filters, such as using interferometric architecture^15,19^, programmable pulse shaping^16,20^, or Talbot-based signal processor^22^. Although our soliton-based RF filters are much simpler than previous microcomb-based RF filters^15–17^ as we eliminate the use of two bulky pulse shapers, the form factor is not yet as compact as their electrical counterparts. Nevertheless, the basic components of our filters can be integrated^7^. The recent advancements on the integration between laser chip and microresonator^28,29^, as well the possibility to replace the SMF with a highly dispersive integrated waveguide^7^, can be further connected to the current work for miniaturization. To conclude, our work significantly reduces the system complexity, size, and cost of the microcomb-based RF filters, while preserving their wide reconfigurability. The proposed schemes set as a stepping stone for chip-scale, cost-effective, and widely reconfigurable microcomb-based RF filters.

## Methods

### Experimental setup

A C-band tunable CW laser is amplified by an Erbium-doped fiber amplifier (EDFA) with amplified spontaneous emission (ASE) filtered, polarization aligned at the TE mode, and then coupled to the Si_3_N_4_ microresonator for soliton microcomb generation. The input and output coupling of the chip is achieved via lensed fibers of ~30% fiber–chip–fiber coupling efficiency. The soliton microcombs are initiated by scanning the pump over the resonances, with the assistance of an arbitrary function generator^27^. The residual pump of generated microcombs is then filtered by a tunable fiber Bragg grating, while a circulator is inserted in between to avoid back-reflection. 10% of light is tapped to an optical spectrum analyzer to record the microcomb spectra. The other 90% of the light is amplified, and polarization managed, before sending to a 30 GHz bandwidth MZM. RF signals from the VNA are applied to the MZM in DSB modulation format. The modulated spectra are then propagated through a spool of 4583.8m SMF to acquire dispersive delays, and finally beats at an 18 GHz PD to convert the signals back to the RF domain. The length of SMF is measured by a commercial optical time-domain reflectometer.

For the system demonstration, a 12 GSa/s arbitrary waveform generator (AWG) is used to prepare the input RF signals. A 40 Mb/s PSK signal modulated at 1.96 GHz tone and 20 Mb/s PSK signal modulated at 3.05 GHz tone are generated separately from the two channels of the AWG. After adding the two streams of signals in a combiner, the composite signal is then sent through the TSM-based RF filters, tuned at 1.96 and 3.05 GHz, respectively. The output spectra are measured by an electrical spectrum analyzer, while the waveforms are measured using a high-speed real-time oscilloscope.

### Si_3_N_4_ microresonator

The Si_3_N_4_ microresonator used in the experiment is a ring structure with a radius of 217 μm. Its waveguide cross-section (width × height) is made to be 1500 nm × 750 nm. The microresonator is coupled with a bus waveguide, which possesses the same cross-section as the ring to realize high coupling ideality^40^. To achieve critical coupling for the resonances, the gap distance between the ring and bus waveguide is designed to be 690 nm. In our experiment, the pumped resonances are around 1556 nm, where both the intrinsic linewidths and coupling strengths are approximately 20 MHz (see Supplementary Note 2). With respect to the reference resonance of *ω*_0_/2*π* = 192.8 THz, the dispersion parameters of microresonator are measured: FSR of microresonator *D*_1_/2*π* ≈ 103.9 GHz, second-order dispersion term *D*_2_/2*π* ≈ 1.28 MHz, and negligible third-order dispersion term ${D}_{3}/2\pi \sim {\mathcal{O}}(1)\,{\rm{kHz}}$ (see Supplementary Note 2).

### LLE simulation

The simulation performed in this work is based on the perturbed LLE model:

3 $$\frac{\partial A(\phi ,t)}{\partial t}=-\Bigg(\frac{\kappa }{2}+j({\omega }_{0}-{\omega }_{p})\Bigg)A(\phi ,t)+j\frac{{D}_{2}}{2}\frac{{\partial }^{2}A(\phi ,t)}{\partial {\phi }^{2}}+jg| A(\phi ,t){| }^{2}A(\phi ,t)+\sqrt{{\kappa }_{{\rm{ex}}}}{s}_{{\rm{in}}},$$

where *A*(*ϕ*, *t*) is the temporal envelope of the intracavity field. *κ* = *κ*_ex_ + *κ*_0_ is the total cavity loss rate, where *κ*_ex_ is the coupling rate, and *κ*_0_ is the internal loss rate. *ω*_0_ and *ω*_p_ denote the angular frequencies of the pumped resonance and the CW pump laser, respectively. *g* is the Kerr frequency shift per photon, defined as $g=\hslash {\omega }_{0}^{2}c{n}_{2}/{n}_{0}^{2}{V}_{{\rm{eff}}}$, where *n*_0_ is the effective group refractive index, *n*_2_ is the nonlinear optical index, and *V*_eff_ is the effective mode volume. *D*_2_ corresponds to the second-order dispersion term, and ∣*s*_in_∣^2^ is the pump power. To involve the AMX effect, an additional frequency detuning *Δ*_*k*_ is introduced at the *k-*th mode, so that the frequency of the *k-*th mode becomes *ω*_*k*_ = *ω*_0_ + *D*_1_*k* + *D*_2_*k*^2^/2 + *Δ*_*k*_ (see Supplementary Note 2). Here in simulation, the dispersion is limited to *D*_2_, and the Raman and thermal effects are not taken into account. According to the dispersion measurement and the generated microcomb spectra, the parameters for the AMX in the simulation are set as *k* = 15 and *Δ*_*k*_/2*π* = 100 MHz, enabling the modulation of the CW intracavity background for the trapping of soliton temporal positions. Note that the strength of the AMX here is estimated to introduce the regularizability of solitons, but without disturbing their formations^36^. Other parameters used in the simulation are retrieved from the characterization, that is, *D*_1_/2*π* = 103.9 GHz, *D*_2_/2*π* = 1.28 MHz, *κ*_ex_/2*π* = *κ*_0_/2*π* = *κ*/4*π* = 20 MHz.

The simulation of the stability chart is obtained by initializing the numerical model with single-soliton solution at various pump power and detuning conditions^36^. Four different states are found: MI, breathers, spatio-temporal and transient chaos, and stable DKS states. The threshold pump power, separating the PSC and stochastic DKS formations, is estimated from both the simulation and experimental conditions. For the TSM simulation, the numerical model is operated under standard CW laser pump scanning from blue-detuned to red-detuned side, until it reaches the stable TSM states. All the possible angles of TSM are recorded. To test the robustness of the TSM azimuthal angle, the model is initialized with one of the exact two-soliton solution, but deliberately perturbed by 10.0° angle deviation. Two solitons are gradually re-stabilized at its original azimuthal angle after a period of free running.

### RF filter response fitting

As the generated microcomb spectra are broader than the amplifying bandwidth of EDFA, we also measured the optical spectra after the EDFA, in order to extract the TDL filter tap weights. The third-order dispersion *β*_3_ of SMF is taken into account for the fitting of RF responses, which can be formulated as^20^:

4 $$H({f}_{{\rm{RF}}}) \sim 	\, \mathop{\sum }\limits_{k}^{}{p}_{k}\cos (2{\pi }^{2}{\phi }_{2}{f}_{{\rm{RF}}}^{2}+4{\pi }^{3}{\phi }_{3}k{f}_{m}^{2}{f}_{{\rm{RF}}})\exp (j4{\pi }^{2}{\phi }_{2}k{f}_{m}{f}_{{\rm{RF}}}\\ 	\,+j4{\pi }^{3}{\phi }_{3}{k}^{2}{f}_{m}^{2}{f}_{{\rm{RF}}}+j\frac{4}{3}{\pi }^{3}{\phi }_{3}{f}_{m}^{3}),$$

where *ϕ*_3_ = −*β*_3_*L*. In accordance with typical values of SMF dispersion, *β*_2_ = −20.2 ps^2^/km and *β*_3_ = 0.117 ps^3^/km at 1550 nm are estimated for all the above fittings of RF filters. The simulation results are in excellent agreement with experimental RF filter responses.

### TSM spectral fitting

First, we extract the power of each comb mode of experimental TSM spectra, and indexed them with respect to the pump mode. Pump mode is rejected and amplitude rescaling is considered as a fitting parameter. Note that the amount of spectral redshift due to Raman effect is also estimated in fitting, by displacing the center of sech^2^ soliton spectra from the pump comb mode. Then, the rescaling parameter and redshift are estimated to best fit the experimental data with the TSM spectral power equation (see Supplementary Note 1), thereby retrieving the azimuthal angle *α* between two solitons. Excellent match between simulations and experimental spectra are obtained.

## Supplementary information

Supplementary Information

## Data Availability

The data used to produce the results of this paper are available at 10.5281/zenodo.3902646.

## Code availability

The code used to produce the results of this paper is available at 10.5281/zenodo.3902646.
