# Supplementary material for: Reconfigurable radiofrequency filters based on versatile soliton microcombs
Source: Nat Commun. 2020 Sep 1;11:4377. doi: 10.1038/s41467-020-18215-z (PMC7462991; doi:10.1038/s41467-020-18215-z)
Supplement: Supplementary file 1 — Supplementary Information [file 41467_2020_18215_MOESM1_ESM.pdf]

Supplementary Information for:
Reconfigurable radiofrequency filters based on versatile soliton microcombs

Hu et al.

Supplementary Note 1. Derivation of RF filter responses

The responses of comb based RF filters can be described by the discrete Fourier transform (FT) of their underlying frequency comb intensity[1]:

$$H(f_{\mathrm{RF}}) \sim \cos(2\pi^2\phi_2 f_{\mathrm{RF}}^2)\sum_k p_k \exp(j4\pi^2\phi_2 k f_m f_{\mathrm{RF}}) \tag{1}$$

where p_k denotes the power of each comb line, $\phi_2 = -\beta_2 L$ is the product of the second-order dispersion β_2 of the dispersive element and its length L, and f_m is the comb line spacing. $f_{\mathrm{FSR}} = 1/(2\pi\phi_2 f_m)$ is the FSR of the RF filters. The exact RF filter responses are obtained by substituting the microcomb spectral profile into Eq. (1). For single-soliton microcomb, the optical field is given by:

$$E(t) \sim \mathrm{sech}(\frac{t}{T_0}) \otimes \sum_{n=-\infty}^{\infty} \delta(t - nT) \tag{2}$$

where T_0 and $T = 1/f_m$ are the soliton pulse width and period, respectively. Taking the FT of Eq. (2), the single soliton spectrum is derived as:

$$\widetilde{E}(f) \sim \mathrm{sech}(\pi^2 T_0 f)\sum_{k=-\infty}^{\infty} \delta(f - kf_m) \tag{3}$$

where f is the frequency offset between the comb mode and pump frequency. This leads to the power of each comb line, or equivalently filter tap weights, being $p_k \sim \mathrm{sech}^2(\pi^2 k T_0/T)$. $k \in Z$ is the mode index with respect to the center comb line. Disregarding the envelope term of Eq. (1) for the moment, we can rewrite the summation part using Poisson summation formula:

$$\sum_{k=-\infty}^{\infty} p_k e^{j2\pi k\frac{f_{\mathrm{RF}}}{f_{\mathrm{FSR}}}} = f_{\mathrm{FSR}} P(f_{\mathrm{RF}}) \otimes \sum_{n=-\infty}^{\infty} \delta(f_{\mathrm{RF}} - nf_{\mathrm{FSR}}) \tag{4}$$

where $P(f_{\mathrm{RF}})$ is the FT of the generalized form of p_k, at which the mode index k is substituted by an arbitrary variable x, times a factor f_{FSR}:

$$P(f_{\mathrm{RF}}) = \int \mathrm{sech}^2(\frac{\pi^2 T_0 f_{\mathrm{FSR}}}{T}x)e^{j2\pi f_{\mathrm{RF}} x}dx \sim \frac{2\frac{T}{T_0}\frac{f_{\mathrm{RF}}}{f_{\mathrm{FSR}}}}{\sinh(\frac{T}{T_0}\frac{f_{\mathrm{RF}}}{f_{\mathrm{FSR}}})} \equiv G(f_{\mathrm{RF}}) \tag{5}$$

where the FT of sech-squared function can be found using the residue theorem[2], and is defined as $G(f_{\mathrm{RF}})$. The single-soliton based RF filter response is derived by substituting $G(f_{\mathrm{RF}})$ back to Eq. (4) and incorporating the envelope term.

Two-soliton microcomb (TSM) based RF filter responses can be obtained in a similar manner. Assume two solitons are of identical amplitude and pulse width:

$$E(t) \sim [\mathrm{sech}(\frac{t}{T_0}) + \mathrm{sech}(\frac{t-\frac{\alpha T}{2\pi}}{T_0})] \otimes \sum_{n=-\infty}^{\infty} \delta(t-nT) \tag{6}$$

where α is the azimuthal angle between two solitons, expressed in radian. The TSM spectrum can be found by FT of Eq. (6):

$$\widetilde{E}(f) \sim \mathrm{sech}(\pi^2 T_0 f)(1+e^{-j\alpha k}) \sum_{k=-\infty}^{\infty} \delta(f-kf_m) \tag{7}$$

We then obtain the filter tap weights as:

$$p_k \sim \mathrm{sech}^2(\pi^2 k T_0/T)(2+2\cos(\alpha k)) \tag{8}$$

where $k \in Z$ is the comb mode index relative to the center mode. Inserting Eq. (8) back to Eq. (4) derives:

$$\sum_{k=-\infty}^{\infty} p_k e^{j2\pi k \frac{f_{\mathrm{RF}}}{f_{\mathrm{FSR}}}} \sim (2G(f_{\mathrm{RF}}) + G(f_{\mathrm{RF}} - \frac{\alpha}{2\pi} f_{\mathrm{FSR}}) + G(f_{\mathrm{RF}} + \frac{\alpha}{2\pi} f_{\mathrm{FSR}})) \otimes \sum_{n=-\infty}^{\infty} \delta(f_{\mathrm{RF}} - n f_{\mathrm{FSR}}) \tag{9}$$

Thus, incorporating the envelope term would eventually lead to the RF filter responses of TSM spectra.

Besides, we can also derive the RF filter bandwidth from Eq. (5), which is approximated by $2.982\frac{T_0}{T} f_{\mathrm{FSR}}$. It is proportional to the soliton pulse width and the RF filter FSR, as well as the repetition-rate of the underlying microcomb.

Supplementary Note 2. Si_3N_4 microresonator characterization

Supplementary Figure 1a shows a picture of Si_3N_4 microresonator chips used in the experiment. Accurate calibrated transmission spectrum of the microresonator is obtained using frequency-

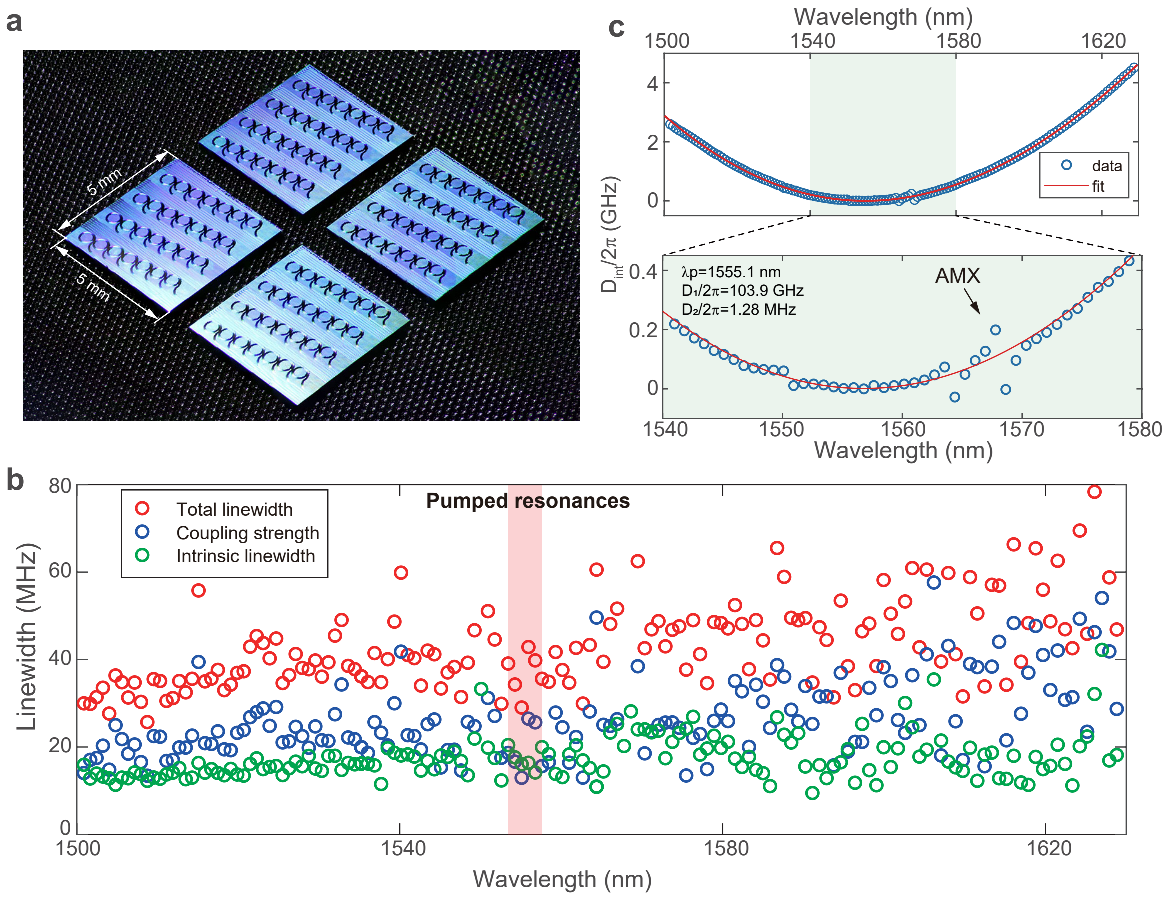

Supplementary Figure 1: Si_3N_4 microresonator characterization. (a) Optical image of the Si_3N_4 microresonator chips. (b) Total linewidth, coupling strength, and intrinsic linewidth of each resonance in the TE_{00} mode family. The shaded area corresponds to the resonances pumped in the experiment. (c) Top: Measured integrated GVD ($D_{int}/2\pi$) of the TE_{00} mode family in the microresonator, with respect to the resonance of 1555.1 nm; Bottom: zoom-in of integrated GVD region between 1540 nm and 1580 nm. Dominant AMX is observed around wavelength region of 1565 nm.

comb-assisted diode laser spectroscopy[3], covering the wavelength region from 1500 nm to 1630 nm. Supplementary Figure 1b illustrates the detailed properties of the resonances, i.e. intrinsic linewidths $\kappa_0/2\pi$, coupling strengths $\kappa_{\mathrm{ex}}/2\pi$, as well as the total linewidths $\kappa/2\pi = (\kappa_0 + \kappa_{\mathrm{ex}})/2\pi$ of the TE_{00} mode family, which are extracted from the fittings of calibrated transmission spectrum. The shaded area of Supplementary Figure 1b denotes the experimentally accessed resonances. All these resonances show intrinsic linewidths $\kappa_0/2\pi \approx 20$ MHz, indicating the Q factors to be around 10^7. Besides, the coupling strengths of the pumped resonances are similar to their intrinsic linewidths,

implying these resonances near critical coupling condition.

Then, the integrated GVD of the TE_{00} mode family are extracted by identifying the precise frequency of each TE_{00} resonance, as shown in the upper part of Supplementary Figure 1c. It can be formulated as:

$$D_{\text{int}}(\mu) = \omega_\mu - (\omega_0 + D_1\mu) = D_2\mu^2/2 + D_3\mu^3/6 + ... \tag{10}$$

where $D_1/2\pi$ is the FSR of microresonator, and $D_n(n \in N_+ \mid n \geq 2)$ correspond to the n-th order dispersion coefficients. $D_{\text{int}}(\mu)$ is defined as the deviation of the μ-th resonance frequency $\omega_\mu/2\pi$ from the equidistant frequency grid, constructed from the FSR around the reference resonance frequency $\omega_0/2\pi$. Here, the reference resonance is chosen at $\omega_0/2\pi = 192.8$ THz (i.e. $\lambda_0 = 1555.1$ nm). The retrieved dispersion terms are $D_1/2\pi \approx 103.9$ GHz, $D_2/2\pi \approx 1.28$ MHz, and $D_3/2\pi \sim \mathcal{O}(1)$ kHz. Note that operating at anomalous dispersion ($D_2 > 0$) is a prerequisite for soliton formation. The bottom part of Figure 1c shows the zoom-in view of the integrated GVD between 1540 nm and 1580 nm. Several resonance frequency deviations are clearly observed in the measured GVD profile, and the most dominant AMX is found around the wavelength region of 1565 nm.

Supplementary Note 3. PSC and TSM soliton steps

Supplementary Figure 2 depicts different soliton step formations by pumping the resonance of 1555.1 nm under and above the threshold pump power level. The soliton step is manifested from the transmission of the microresonator by scanning the CW pump laser over the resonance. When the pump power is around 15 mW, only a single PSC step is formed, and a soliton number of 4 can be deduced from the depth of the step. However, if the power of scanning CW laser is increased to around 50 mW, both two-soliton and single-soliton steps would appear. The distinct soliton steps clearly indicate two different soliton generation regimes, and are consistent with experimentally generated microcomb spectra.

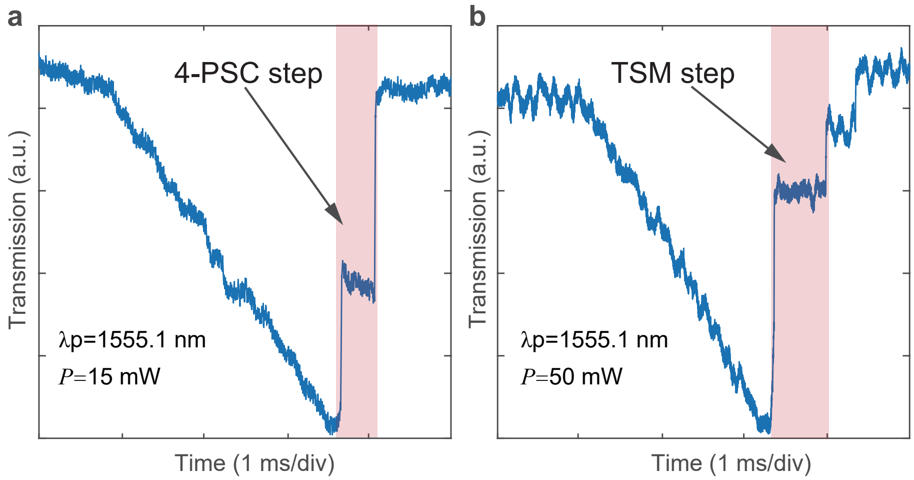

Supplementary Figure 2: Soliton steps for PSC and TSM formations. Transmission curves are obtained by scanning a laser across the resonance below (a) and above (b) the threshold pump power, at resonance of $1555.1\ \mathrm{nm}$. The shaded areas in (a) and (b) correspond to 4-PSC step and TSM step, respectively.

Supplementary Note 4. TSM based RF filters of different resonances

We also investigate TSM based RF filters from different pumped resonances. Supplementary Figure 3a shows the experimentally synthesized RF filters centered at $1.83\ \mathrm{GHz}$, $2.83\ \mathrm{GHz}$, $3.94\ \mathrm{GHz}$, $4.95\ \mathrm{GHz}$, $6.00\ \mathrm{GHz}$, and $7.06\ \mathrm{GHz}$ from the resonance of $1555.1\ \mathrm{nm}$. While pumped at resonance of $1556.8\ \mathrm{nm}$, the filter passband frequencies are relocated at $0.90\ \mathrm{GHz}$, $2.07\ \mathrm{GHz}$, $3.23\ \mathrm{GHz}$, $4.52\ \mathrm{GHz}$, $5.55\ \mathrm{GHz}$, and $6.90\ \mathrm{GHz}$ (Supplementary Figure 3b). By exploring adjacent resonances of $1556.0\ \mathrm{nm}$, the maximum grid of TSM based RF filters is reduced to be less than $1\ \mathrm{GHz}$.

The passband frequency shifts of RF filters arise from the variation of their underlying two-soliton azimuthal angles, which are experimentally retrieved from their TSM spectra and compared to numerical simulation (Supplementary Figure 4a). In the simulation, the AMX position is varied from 14-th to 16-th away from the pump of the same strength, to resemble the change of resonances in line with experimental condition. The background modulation period is then modified according to the relative distance between the pump mode and the AMX. Besides, towards large relative soliton angle, the modification of its value through pumped resonance also becomes more prominent. This effect is simply due to the accumulated periodicity difference, and is clearly observed

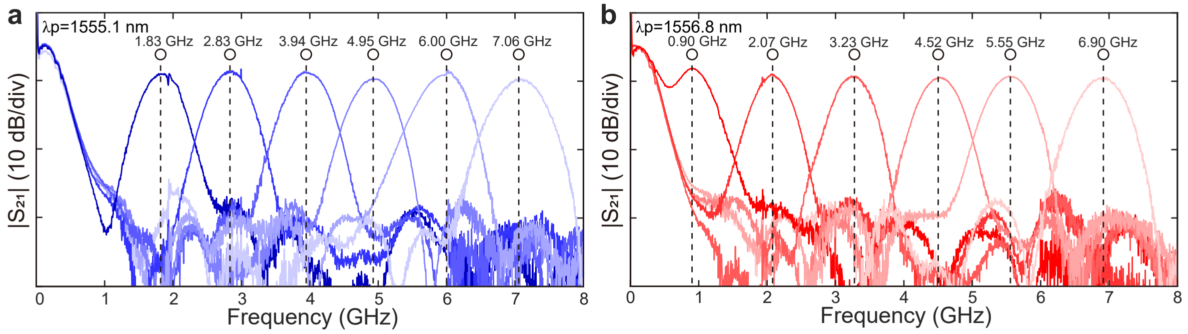

Supplementary Figure 3: TSM based RF filter responses at resonances of 1555.1 nm and 1556.8 nm. (a) RF filters centered at 1.83 GHz, 2.83 GHz, 3.94 GHz, 4.95 GHz, 6.00 GHz, and 7.06 GHz are obtained at resonance of 1555.1 nm. (b) RF filters centered at 0.90 GHz, 2.07 GHz, 3.23 GHz, 4.52 GHz, 5.55 GHz, and 6.90 GHz are obtained at resonance of 1556.8 nm.

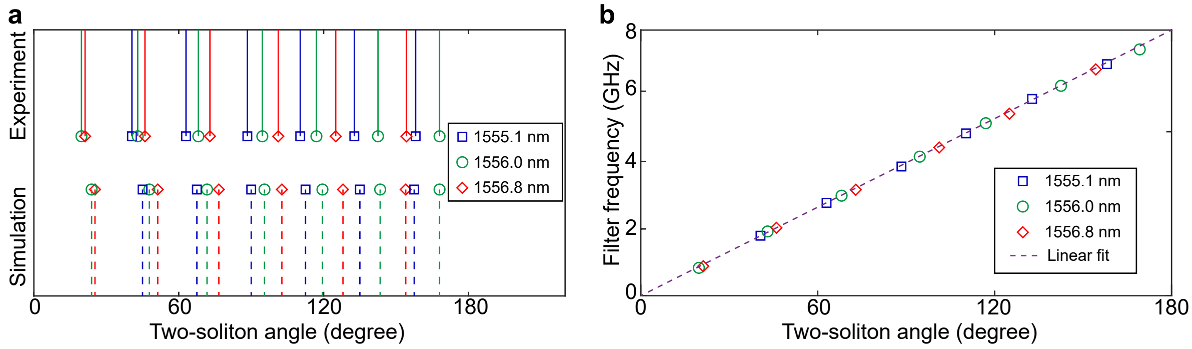

Supplementary Figure 4: Experimental and simulated azimuthal angles of TSM spectra. (a) Experimentally retrieved (solid lines, top) and simulated (dashed lines, bottom) two-soliton angles at resonances of 1555.1 nm, 1556.0 nm, and 1556.8 nm. (b) The synthesized RF filter frequencies versus their underlying two-soliton angles retrieved from TSM spectra.

in both measured and simulated results. Supplementary Figure 4b illustrates the relation between the experimental RF passband frequencies and their corresponding TSM azimuthal angles. A good linear approximation confirms well the operation principle of proposed TSM based RF filters.

Supplementary Note 5. Link performances of the microcomb based RF filters

We also characterize and optimize the gain and noise figure of our microcomb based RF filters. We measure in particular the RF filter centered around $4\ \mathrm{GHz}$ based on 4-PSC, while the performances as well as optimization technique are similar for other cases. An RF amplifier is used to lift the noise floor of the electrical spectrum analyzer (ESA) (set at $1\ \mathrm{Hz}$ resolution bandwidth) when measuring the filter noise figure. And the noise figure is assessed at the center frequency of the filter which is of the primary interest. Since the link gain depends quadratically on the optical power, we use a photodetector with high power handing capability (maximum input power $\sim +16\ \mathrm{dBm}$, responsivity $\sim 0.6\ \mathrm{A/W}$) in the link experiment.

It is well known that the DC bias point of the MZM plays an important role in determining the filter's gain and noise figure. The filter gain is maximized when the MZM is biased at quadrature. However, when the bias is gradually decreased to the minimum transmission point (MITP), although the gain drops, the noise actually reduces faster than the gain, which is known as the low-biasing technique[4]. Thus, we vary the MZM DC bias while keeping the same optical power at the MZM input around $150\ \mathrm{mW}$ (limited by its maximum input power), and we measure the gain and noise figure of the filter as shown in Supplementary Figure 5a. The DC bias is normalized here, where 0, 0.5, 1 correspond to bias points at MATP (maximum transmission point), quadrature, and MITP, respectively. Indeed, when we tune the bias from quadrature towards MITP, both the filter gain and noise figure are decreased. Note that we were not able to measure the noise figure values at very low bias point close to MITP, as the noise floor there becomes comparable with the displayed noise level[5]. We could obtain RF filter with loss of $24.7\ \mathrm{dB}$ and noise figure of $27.7\ \mathrm{dB}$.

The loss of the RF filter due to the low-biasing can be simply compensated by another EDFA at the output of the MZM[5]. We carry out the filter gain and noise figure characterization in the same way, which is shown in Supplementary Figure 5b. In this scenario, the optical power is limited by the maximum input power to the photodetector, where we operated at around $25\ \mathrm{mW}$. We can see that the filter gain increases first with the DC tuned from quadrature towards MITP, and suddenly

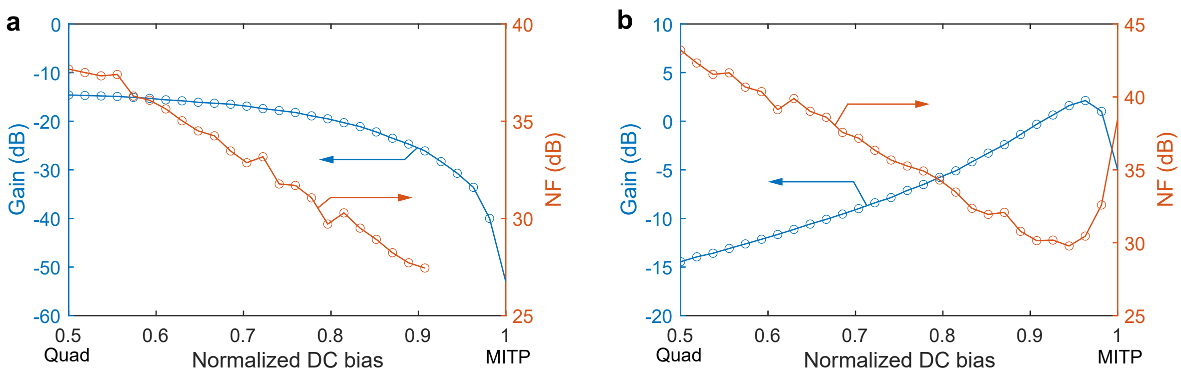

Supplementary Figure 5: Gain and noise figure of the RF filter based on 4-PSC. RF filter gain and noise figure (NF) versus MZM normalized bias (a) without EDFA at the MZM output (b) with EDFA at the MZM output.

decreases when it is very close to MITP. This is because the power of modulated sidebands (signals) are very small at the beginning, the filter gain increases when the power between the carriers and signals become more balanced, as the gain relates to the product of the signals and carriers power. And the gain reaches peak when the power of them become comparable. After that, the filter gain drops quickly due to the weak carriers as very close to the null point. In terms of noise figure, it reduces at the beginning due to the low-biasing, but increases dramatically afterwards when the input power to the EDFA is too low, which tends to amplify more noise instead of the carriers and signals. Overall, we could achieve positive RF filter gain ($+1.6$ dB) with noise figure of 29.8 dB.

The further optimization of link performance can be envisioned using MZM with lower half-wave voltage (V_π) and higher maximum input power, EDFA with lower noise figure, and photodetector with higher responsivity and higher power handling capability. We note that MZM with V_π as low as 1.4 V[6] is now feasible by integrated solution, which is much lower than the MZM we used. The optimal condition will use the low-biasing technique and bypass the EDFA after the MZM, while still achieving relatively high RF gain.

Supplementary Note 6. Discussion on the all-optical reconfiguration of the RF filters

The modification of our RF filters rely fundamentally on the inherent stable and accessible DKS states. In terms of PSC microcombs, different PSC states are accessed by pumping at different resonances under the threshold power. The maximum soliton number that can be sustained in the microresonator is roughly estimated as $\sqrt{\kappa/D_2}$[7]. PSC with a higher number of pulses would lead to the interaction between them. By substituting the total linewidths of the pumped resonances, the maximum PSC number in our chip is estimated around 5. This provides a good approximation as we can achieve PSC numbers from 2 to 4 experimentally. We also need to point out here that not every PSC state below this predicted value is easily generated, especially for a large maximum PSC number[7].

For the TSM spectra, the two solitons are locked to a few relative angles according to the AMX profile. Since the route to TSM undergoes chaotic region, the soliton number as well as relative distribution remain stochastic, unlike PSC formation. However, these different TSM seem to be roughly equiprobable, and all TSM states in this letter are obtained within 25 times trial in total for each resonance. We also carry out experimental statistical test (in total 100 times trial) on the probability of TSM relative angles, as seen in the Supplementary Figure 6. All the soliton angles are accessed with similar probability. Since the possible configurations of two solitons are relatively small in our case, we could simply implement the laser scanning multiple times until we get the desired angle. A pulse triggering technique could be envisioned in the future to deterministically switch to the TSM with targeted azimuthal angle, as has been adopted in fiber cavity[8]. Indeed, a recent theoretical work[9] has proved the feasibility of adapting this technique to the microresonator. Basically, it uses a single pulse or pulse trains to trigger deterministically the soliton states, while the main interest in that study is on the single soliton. In a similar manner, when replacing the initial single pulse by two pulses of controlled delay, the deterministic generation of two soliton with the corresponding separation can be achieved.

Additionally, another possible scheme is to first generate TSM state and then tune the soliton

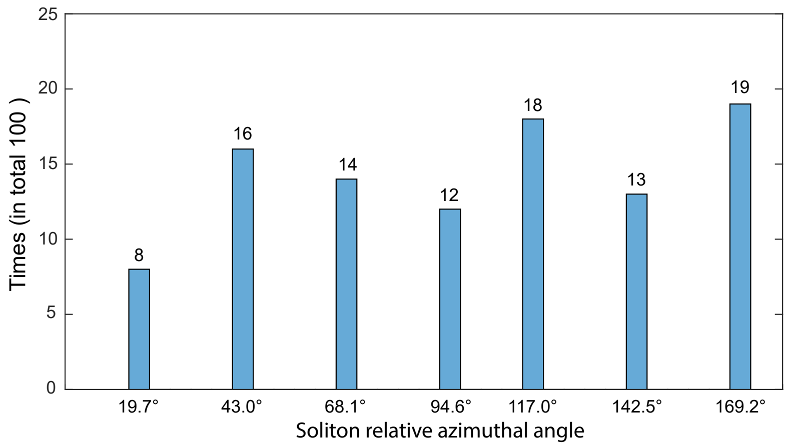

Supplementary Figure 6: Probability distribution of relative soliton angles in the TSM states. The TSM states are experimentally obtained 100 times at resonance of 1556.0 nm.

angle to the desired value. This may be achieved via the modification of the intracavity background. Since the solitons are always trapped to the certain positions of the modulated background, the continuous change of the background modulation will push the solitons to follow the new background. Thus, by changing the AMX position, via heating in a coupled ring structure[10] for example, the background modulation induced by the beating of pump laser and AMX will be modified accordingly. Moreover, the background modulation can also be formed using dichromatic pump, in which the beating between two pump lasers of different wavelengths defines the background modulation properties[11]. Under appropriate conditions, the relative position of two solitons may be controlled via adjusting the wavelengths and power of two pumped lasers.

Supplementary References

1. Zhu, X., Chen, F., Peng, H. & Chen, Z. Novel programmable microwave photonic filter with arbitrary filtering shape and linear phase. *Optics express* **25**, 9232–9243 (2017). URL https://doi.org/10.1364/oe.25.009232.

2. Haus, H. A. *Electromagnetic noise and quantum optical measurements* (Springer Science & Business Media, 2012). URL https://doi.org/10.1007/978-3-662-04190-1.

3. Liu, J. *et al.* Frequency-comb-assisted broadband precision spectroscopy with cascaded diode lasers. *Optics letters* **41**, 3134–3137 (2016). URL https://doi.org/10.1364/ol.41.003134.

4. Marpaung, D. *et al.* Integrated microwave photonics. *Laser & Photonics Reviews* **7**, 506–538 (2013). URL https://doi.org/10.1002/lpor.201200032.

5. Liu, Y., Marpaung, D., Choudhary, A., Hotten, J. & Eggleton, B. J. Link performance optimization of chip-based si 3 n 4 microwave photonic filters. *Journal of lightwave technology* **36**, 4361–4370 (2018). URL https://doi.org/10.1109/jlt.2018.2842203.

6. Wang, C. *et al.* Integrated lithium niobate electro-optic modulators operating at cmos-compatible voltages. *Nature* **562**, 101–104 (2018). URL https://doi.org/10.1038/s41586-018-0551-y.

7. Karpov, M. *et al.* Dynamics of soliton crystals in optical microresonators. *Nature Physics* 1–7 (2019). URL https://doi.org/10.1038/s41567-019-0635-0.

8. Leo, F. *et al.* Temporal cavity solitons in one-dimensional kerr media as bits in an all-optical buffer. *Nature Photonics* **4**, 471 (2010). URL https://doi.org/10.1038/nphoton.2010.120.

9. Kang, Z. *et al.* Deterministic generation of single soliton kerr frequency comb in microresonators by a single shot pulsed trigger. *Optics express* **26**, 18563–18577 (2018). URL https://doi.org/10.1364/oe.26.018563.

10. Xue, X. *et al.* Normal-dispersion microcombs enabled by controllable mode interactions. *Laser & Photonics Reviews* **9**, L23–L28 (2015). URL https://doi.org/10.1002/lpor.201500107.

11. Taheri, H., Matsko, A. B. & Maleki, L. Optical lattice trap for kerr solitons. *The European Physical Journal D* **71**, 153 (2017). URL https://doi.org/10.1140/epjd/e2017-80150-6.
